# Interaction between *CYP1A1* gene polymorphism and environment factors on risk of endometrial cancer

**DOI:** 10.1265/ehpm.24-00007

**Published:** 2024-10-10

**Authors:** Jian Xu, Cheng Tan

**Affiliations:** 1 Department of Oncology, the second people's hospital of Nantong, Affiliated Nantong Rehabilitation Hospital of Nantong University, Nantong City, Jiangsu Province, China, 226002; 2 Department of Radiotherapy, Nantong Tumor Hospital, Tumor Hospital Affiliated to Nantong University, Nantong City, Jiangsu Province, China, 226300

**Keywords:** Abdominal obesity, *CYP1A1*, Endometrial cancer, Interaction, Polymorphism

## Abstract

**Background:**

The purpose of this study was to investigate the impact of single nucleotide polymorphisms (SNPs) of the *CYP1A1* gene and the gene-environment interaction on the susceptibility to endometrial cancer in Chinese women.

**Method:**

Logistic regression was performed to investigate the association between the four SNPs of the *CYP1A1* gene and the risk of endometrial cancer. Generalized multifactor dimensionality reduction (GMDR) was employed to analyze the gene-environmental interaction.

**Results:**

A total of 934 women with a mean age of 61.7 ± 10.5 years were selected, including 310 endometrial cancer patients and 624 normal controls. The frequency of rs4646421- T allele was higher in endometrial cancer patients than normal controls, the T allele of rs4646421 was 28.1% in endometrial cancer patients and 21.0% in normal controls (*p* < 0.001). Logistic regression analysis showed that the rs4646421 - T allele was associated with increased risk of endometrial cancer, OR (95% CI) were 1.52 (1.11–1.97) and 1.91 (1.35–2.52), respectively. GMDR analysis found a significant two-locus model (p = 0.0107) involving rs4646421 and abdominal obesity (defined by waist circumference), indicating a potential gene–environment interaction between rs4646421 and abdominal obesity. Abdominal obese subjects with rs4646421- CT or TT genotype have the highest risk of endometrial cancer, compared to non-abdominal obese subjects with the rs4646421- CC genotype, the OR (95%CI) was 2.23 (1.62–2.91).

**Conclusions:**

Both the rs4646421- T allele and the interaction between rs4646421 and abdominal obesity were associated with increased risk of endometrial cancer.

## Introduction

Endometrial cancer is a hormone-dependent malignant tumor that occurs in the endometrium. It is common in perimenopausal and postmenopausal women, and the incidence rate of endometrial cancer was increasing year by year around the world [1, 2]. According to statistics in 2022, there were approximately 65,950 new endometrial cancer cases and 12,550 deaths due to endometrial cancer [3]. In addition, the 5-year relative survival rate of advanced endometrial cancer cases is relatively low, about 17.1% [4]. In recent years, the overall incidence of endometrial cancer in China has shown an upward trend, and the incidence rate of endometrial cancer of young women under 40 years old in rural areas is growing rapidly [5]. In the clinic, 75% to 90% of endometrial cancer were type I [6]. Although the pathogenesis of endometrial cancer has not been fully understood, endometrial cancer susceptibility could be influenced by multiple genetic and molecular characteristics [7]. Currently, studies [8, 9] have shown that obesity, hypertension, diabetes, and DNA damage may be related to pathogenesis.

The *CYP1A1* gene mainly encodes the enzyme cytochrome P450 1A1 (CYP1A1), which has aryl hydrocarbon hydroxylase activity [10], can convert polycyclic aromatic hydrocarbons into aryl epoxide carcinogens [11]. The *CYP1A1* gene, located on chromosome 15q24.1, consists of 7 exons and 512 residues, and is mainly expressed in extrahepatic tissues. The CYP1A1 gene polymorphism may reduce 2-OH estrogen, increasing the opportunity to exposure to 4-OH estrogen, thus increasing the risk of endometrial cancer [12]. In the past decades, several epidemiological studies have reported the relationship of *CYP1A1* gene single nucleotide polymorphism (SNP) with breast cancer [13], cervical cancer [14, 15], and endometrial cancer [16–18]. However, the results on the relationship between *CYP1A1* and the risk of endometrial cancer were inconsistent or even contradictory. In addition, no study was performed to evaluate the impact of synergistic effect between *CYP1A1* gene and environmental factors on risk of endometrial cancer in the Chinese population. Therefore, we conducted this study to examine the impact of four SNPs (rs4646421, rs4646903, rs4646422, and rs1048943) of *CYP1A1* gene, and the interaction between *CYP1A1* gene and environmental factors on the susceptibility of endometrial cancer in Chinese women.

## Methods

### Subjects

All participants, including endometrial cancer patients and normal controls, were consecutively recruited between June 2015 and July 2020 from the second people’s hospital of Nantong and the Nantong tumor hospital. Endometrial cancer patients were selected from those patients diagnosed in Nantong tumor hospital. All patients were not treated with chemotherapy, radiotherapy, and molecular-targeted drugs and had no history of other tumors. Those with a history of other cancers and any other major disease, including allergies, cardiovascular disease and infections were not enrolled in the case group. The normal controls were selected from those subjects undergoing health examinations in the second people’s hospital of Nantong, and matched with the cases according to age (± 4 years). Finally, a total of 934 women with a mean age of 61.7 ± 10.5 years were selected, including 310 endometrial cancer patients and 624 normal controls. A total of 2–3 ml of peripheral venous blood were collected from all participants. Waist circumference (WC), blood pressure, fasting blood glucose (FPG) were measured and lifestyle information such as smoking, alcohol consumption and others information including medical history, reproductive status, treatment history, number of pregnancies and family history were also collected. Informed consent was obtained from all participants.

### Genomic DNA extraction and genotyping

A total of four SNPs of *CYP1A1* gene were selected according to the following criterion: 1) those SNPs, which have been reported associations with endometrial cancer risk, but were not verified; 2) those SNPs, the minor allele frequency (MAF) of which was greater than 2%. Finally, four SNPs including rs4646903, rs1048943, rs4646422, and rs4646421 were selected for genotyping in the study. According to the instructions of the DNA Blood Mini Kit (Qiagen, Hilden, Germany), 3 ml of EDTA-processed blood samples were collected from all participants, DNA was extracted and store in a −20 °C refrigerator. PCR-based restriction fragment length polymorphism was used for genotyping of the four SNP, and the primers information and description of 4 SNPs were shown in Table 1. A 25 µl reaction mixture that included 1.25 µl SNP Genotyping Assays (20×), 12.5 µl Genotyping Master Mix (2×), 20 ng DNA, and the conditions were as follows: initial denaturation at 95 °C for 10 min, denaturation at 92 °C for 15 s, annealing and extension at 60 °C for 90 s and 50 cycles. Lastly, we randomly selected 10% of the samples for re-genotyping, and we found that the sampling results were 100% consistent with the previous genotyping results.

**Table 1:** Description and primer sequences used for genotyping for 4 SNPs

**SNP names**	**Chromosome**	**Functional Consequence**	**Nucleotide substitution**	**Primer sequences**
rs4646422	15:74722964	Missense	A>G	Forward: 5′-TTCTCTCAGCAGCCACCTCCA-3′ Reverse: 5′-ATCTGCAGCACGTCCCCATACT-3′
**rs4646421**	15:74723851	Intron variant	C> T	Forward: 5′-CCATTTATTCTCTGCTCTCTGGTA-3′ Reverse: 5′-CCCACCACACTTAGGA AAATCA-3′
rs4646903	15:74719300	Downstream variant 500B	T>C	Forward: 5′-ACTCACCCTGAACCCCATTC-3′ Reverse: 5′-GGCCCCAACTACTCAGAGGCT-3′
rs1048943	15:74720644	Missense	A>G	Forward: 5′-CTGTCTCCCTCTGGTTACAGGAAGC-3 Reverse: 5′-TTCCACCCGTTGCAGCAGGATAGCC-3′

### Statistical analysis

SPSS 22.0 software was used for all statistical analysis in this study. For continuous variables with normal distribution, mean and standard deviation (SD) were calculated and student’s t test was used for comparison between cases and controls; for classified variables, percentage was calculated and chi-square test was used for comparison between groups. Furthermore, the Hardy-Weinberg equilibrium test (HWE) was calculated using the SNPstats online software (http://bioinfo.iconologia.net/SNPstats). Logistic regression analysis was used to analyze the relationship between CYP1A1 SNP and endometrial cancer risk, and hierarchical analysis of interaction. Generalized multifactor dimensionality reduction (GMDR) [19] was used to screen the best gene-environment interaction model and the relevant indicators were calculated.

## Results

A total of 934 Chinese Han women were included in this study, with an average age of 61.7 ± 10.5 years, including 310 patients with endometrial cancer and 624 normal controls. Table 2 shows the general demographic and clinical characteristics of endometrial cancer patients and normal controls. The mean age and rates of T2DM and hypertension were not significantly different between the case and control group. The mean WC was significantly higher in endometrial cancer patients than controls. The rates of smoking, alcohol consumption, history of contraceptive use, and number of pregnancies were higher in patients than controls.

**Table 2:** General characteristics of study participants in endometrial carcinoma patients and controls

**Variables**	**Cases group** **(n = 310)**	**Control group** **(n = 624)**	***p*-values**
Age (years)	62.2 ± 13.8	61.4 ± 11.3	0.345
Current smokers, N (%)	22 (7.1)	25 (4.0)	0.042
Alcohol drinking, N (%)	18 (5.8)	17 (2.7)	0.021
WC (cm)	88.9 ± 12.7	85.4 ± 11.6	<0.001
T2DM, N (%)	42 (13.6)	68 (10.9)	0.237
Hypertension, N (%)	65 (21.0)	104 (16.7)	0.108
Contraceptive use history, N (%)	109 (35.2)	141 (22.6)	<0.001
Postmenopausal, N (%)	225 (72.6)	438 (70.2)	0.449
Number of pregnancies			0.017
1	259 (83.5)	533 (85.4)	
2–3	47 (15.2)	91 (14.6)	
>4	4 (1.3)	0 (0)	
Types			
type I	224 (72.3)		
type II	86 (27.7)		
Grades			
G1	161 (51.9)		
G2	77 (24.8)		
G2	72 (23.2)		

Note: means ± standard deviation for age and WC. Comparison for mean and standard deviation (SD) was performed using the student t test, the percentage was calculated for categorical variables including current smokers, regularly alcohol drinking, T2DM, hypertension, contraceptive use history, postmenopausal, number of pregnancies, types and grades, and the chi square test was used for comparison between groups.

The frequencies of rs4646421 minor allele were higher in endometrial cancer patients than normal controls, the frequencies of the T allele of rs4646421 were 28.1% in endometrial cancer patients and 21.0% in normal controls (*p* < 0.001) (Table 3). Logistic regression analysis showed a significant association between variant genotypes of rs4646421 and increased risk of endometrial cancer, after adjustment for age, smoking status, alcohol consumption status, WC, history of contraceptive use, and number of pregnancies. The carriers of homozygous mutant and mutant heterozygote of rs4646421 polymorphism revealed increased endometrial cancer than those with wild-type homozygotes, OR (95%CI) was 1.52 (1.11–1.97) and 1.91 (1.35–2.52), respectively.

**Table 3:** Genotype and allele frequencies of 4 SNPs between endometrial carcinoma cases and controls

**SNPs**	**Genotypes ** **and alleles**	**Frequencies N (%)**		OR (95%CI)*	***p-values***	**H-W test for ** **controls**

		**Patients** **(n = 310)**	**Controls** **(n = 624)**			
rs4646422						
	AA	171 (55.2)	369 (59.1)	Ref		0.266
	AG	121 (39.0)	228 (36.5)	1.21 (0.81–1.67)	0.526	
	GG	18 (5.8)	27 (4.3)	1.43 (0.70–2.17)	0.728	
	A	463 (74.7)	966 (77.4)	Ref		
	G	157 (25.3)	282 (22.6)	1.26 (0.78–1.75)	0.624	
rs4646421						
	CC	161 (51.9)	389 (62.3)	Ref		0.904
	CT	124 (40.0)	208 (33.3)	1.52 (1.11–1.97)	0.0021	
	TT	25 (8.1)	27 (4.3)	1.91 (1.35–2.52)	<0.001	
	C	446 (71.9)	986 (79.0)	Ref		
	T	174 (28.1)	262 (21.0)	1.63 (1.21–2.08)	<0.001	
rs4646903						
	TT	167 (53.9)	364 (58.3)	Ref		0.238
	TC	123 (39.7)	232 (37.2)	1.21 (0.73–1.71)	0.461	
	CC	20 (6.4)	28 (4.5)	1.41 (0.69–2.16)	0.628	
	T	457 (73.7)	960 (76.9)	Ref		
	C	163 (26.3)	288 (23.1)	1.26 (0.70–1.87)	0.537	
rs1048943						
	AA	166 (53.6)	366 (58.7)	Ref		0.330
	AG	116 (37.4)	218 (34.9)	1.40 (0.87–1.92)	0.515	
	GG	28 (9.0)	40 (6.4)	1.63 (0.82–2.46)	0.682	
	A	448 (72.3)	950 (76.1)	Ref		
	G	172 (27.7)	298 (23.9)	1.46 (0.85–2.08)	0.603	

*Adjusted for age, smoking status, alcohol consumption status, WC, contraceptive use history and number of pregnancies.

We employed the GMDR model to evaluate the impact of the gene-gene and gene-environment interaction on endometrial cancer risk, after adjustment for age, smoking status, alcohol consumption status, contraceptive use history, and number of pregnancies. Table 4 summarized the results obtained from the GMDR model analysis. We found a significant two-locus model (*p* = 0.0107) involving rs4646421 and abdominal obesity, indicating a significant gene-environment interaction between rs4646421 and abdominal obesity. Overall, the two-locus model had a cross-validation consistency of 10 of 10 and the testing accuracy of 60.72%.

**Table 4:** Best gene-abdominal interaction models identified by GMDR methods

**Locus no.**	**Best combination**	**Cross-validation consistency**	**Testing balanced accuracy**	p-values*
2	rs4646421* abdominal obesity	10/10	0.6072	0.0100
3	rs4646421* rs4646903 *abdominal obesity	8/10	0.5823	0.1719
4	rs4646421* rs4646903* rs4646422 *abdominal obesity	5/10	0.5451	0.8281
5	rs4646421* rs4646903* rs4646422 * rs1048943* abdominal obesity	8/10	0.5590	0.3770

*Adjusted for age, smoking status, alcohol consumption status, contraceptive use history and number of pregnancies.

To obtain odds ratios and 95%CI for the interaction effects between rs4646421 and abdominal obesity on endometrial cancer risk, we performed hierarchical analysis for interaction between rs4646421 and abdominal obesity using logistic regression. We found that abdominal obese subjects with CT or TT genotype of rs4646421 have the highest risk of endometrial cancer, compared to non-abdominal obese subjects with the rs4646421- CC genotype, OR (95%CI) was 2.23 (1.62–2.91), after adjustment for age, smoking status, alcohol consumption status, contraceptive use history and number of pregnancies (Table 5).

**Table 5:** Interaction between rs4646421 and abdominal obesity on endometrial carcinoma risk

**rs4646421**	**Abdominal obesity**	OR (95% CI)*	***P*-values**
CC	No	1.00	-
CC	Yes	1.63 (1.18–2.09)	0.013
CT or TT	No	1.47 (1.05–1.93)	0.021
CT or TT	Yes	2.23 (1.62–2.91)	<0.001

*Adjusted for age, smoking status, alcohol consumption status, contraceptive use history and number of pregnancies.

## Discussion

In this study, the results indicated that the rs4646421- T allele was associated with increased risk of endometrial cancer in Chinese Han population. Carriers with the rs4646421- T allele have a higher risk of endometrial cancer than those with wild-type homozygotes. Estrogens are mainly distributed on the inner and outer surface of cells and bind to the nuclear binding protein of the estrogen receptor to maintain the high affinity and specificity of target cells. Since the cytochrome P450 (CYP) enzyme is involved in the oxidative metabolism of estrogen, the gene polymorphism related to estrogen biosynthesis and metabolism is potential risk factor for estrogen receptor-positive malignant tumors [20]. The *CYP1A1* gene is one of the main members of the CYP family, which is mainly distributed in extrahepatic tissues and functions mainly as metabolic substrates and exogenous substances [21]. Several studies focused on the relationship between *CYP1A1* gene and several other cancer types, including oral cancer [22], lung cancer [23], larynx cancer [24], breast cancer [13], cervical cancer [14, 15] and endometrial cancer [25–27] risk in different populations. The association of *CYP1A1* gene SNPs with susceptibility to endometrial cancer was not well investigated in different populations, and those previous studies [25–27] concluded inconsistent results. However, few studies were conducted to test the association between SNPs of the *CYP1A1* gene and the risk of endometrial cancer in Han Chinese women. The *CYP1A1* rs1048943 polymorphism is a mutation (base substitution from A to G) in the 3′-untranslated region. Previous studies [12, 24–26] suggested that rs1048943 polymorphism was statistically associated with the risk of endometrial cancer. However, another study performed by Li et al [16] suggested that rs1048943 was not associated with the risk of endometrial cancer in Chinese populations, which was similar with the results obtained from the current study. Previous studies have observed the association between rs4646421 and several cancers, including gastric cancer [28], breast cancer [29], and hepatocellular cancer [30]. However, only one Chinese study [31] has confirmed that rs4646421 polymorphism was associated with increased endometrial cancer, and may be a potential indicator of the endometrial cancer. The biological mechanism of the association between the *CYP1A1* gene and the risk of endometrial cancer is not very clear. Some studies [32, 33] suggested that the activation of the CYP1A1 enzyme is involved in the oxidation of endogenous and exogenous compounds and catalyzes the conversion of polycyclic aromatic hydrocarbons into phenols and epoxides. Some phenols and epoxides can combine with DNA to form adducts, which are finally transformed into carcinogens and diol epoxides. They are resistant to some antitumor drugs through epoxide hydrolase and increase the risk of individual tumors.

The risk of endometrial cancer could be affected by many factors, including genetic factors, environmental factors, and the synergy effect between gene and environmental. Previous studies [34, 35] indicated that obesity was an important risk factor for endometrial cancer. Zaki et al. [36] suggested that abdominal obesity was the most stable obesity index to predict the risk of endometrial cancer in Egyptian women. However, to date, no studies were performed to investigate the impact of interaction between *CYP1A1* gene and abdominal obesity on the risk of endometrial cancer. In this study, the GMDR model was used to evaluate the effects of gene-environment interaction. We found a significant two-locus model involving rs4646421 and abdominal obesity, indicating a potential gene–environment interaction between rs4646421 and abdominal obesity. Those abdominal obese subjects with CT or TT of rs4646421 have the highest risk of endometrial cancer, compared to non-abdominal obese subjects with the rs4646421- CC genotype. Therefore, when genetic and environmental factors not only independently affect the risk of endometrial cancer, but also have a synergistic effect, it means that the impact of genes on the risk of endometrial cancer will be corrected by obesity factors.

The limitations of this study should be considered. Firstly, just four SNPs of *CYP1A1* gene were selected, more SNPs within the *CYP1A1* gene should be included in the future; Secondly, more environmental factors should be included in the interaction analysis. Lastly, the results of this study are based on the Han Chinese population and need to be validated in other different populations or races.

In conclusion, we found that the minor alleles of rs4646421 was associated with increased endometrial cancer risk. In addition, the interaction between rs4646421 and abdominal obesity were also associated with an increased risk of endometrial cancer, which means that the impact of rs4646421 minor allele on endometrial cancer susceptibility could be amplified by abdominal obesity.

## Abbreviations

SNPs: single nucleotide polymorphisms
GMDR: Generalized multifactor dimensionality reduction
FPG: fasting blood glucose
SD: standard deviation
HWE: Hardy-Weinberg equilibrium
WC: Waist circumference
T2DM: type 2 diabetes mellitus
